# Synthesis and Evaluation of Asymmetric Mesoporous PTFE/Clay Composite Membranes for Textile Wastewater Treatment

**DOI:** 10.3390/membranes11110850

**Published:** 2021-11-01

**Authors:** Saida Bousbih, Rihab Belhadj Ammar, Raja Ben Amar, Lasâad Dammak, Fadila Darragi, Emna Selmane

**Affiliations:** 1 Département de Géologie, Faculté des Sciences de Tunis, Université Tunis El Manar, Tunis 2092, Tunisia; bousbih_saida@yahoo.fr (S.B.); fadila.darragi@utm.tn (F.D.); 2 Institut de Chimie et des Matériaux Paris-Est (ICMPE), Université Paris-Est, UMR 7182, CNRS, 2-8 rue Henri Dunant, 94320 Thiais, France; belhadj.rihab25@gmail.com (R.B.A.); dammak@u-pec.fr (L.D.); 3 Laboratoire de Chimie Analytique et d’Électrochimie, Département de Chimie, Faculté des Sciences de Tunis, Université Tunis El Manar, Tunis 2092, Tunisia; emnaselmane@gmail.com; 4 Département de Chimie, Faculté des Sciences de Sfax, Université de Sfax, BP 1141, Sfax 3018, Tunisia

**Keywords:** natural clay, ultrafiltration, mesoporous membrane, PTFE, real textile wastewater

## Abstract

Asymmetric mesoporous composite PTFE membranes wit 40, 50, and 85 wt.% of a clay (kaolin) were fabricated and characterized using a scanning electron microscope equipped with EDX for morphology and elemental analysis. The surface chemistry of the membranes was checked using Fourier transform infrared spectroscopy. The effect of incorporating the clay on the hydrophilicity, permeability, morphology, and antifouling properties of the fabricated membranes was investigated. It was observed that incorporating kaolin particles improved the mechanical properties but decreased the contact angle of the membranes, thereby resulting in an improvement in the membrane permeability. The performance of the three composite UF membranes was evaluated through the treatment of a real textile effluent sample containing indigo dye. The results confirmed that these membranes are effective in the removal of COD, color, and turbidity. Indeed, at a transmembrane pressure of 2.5 bar, almost total removal of the turbidity, COD removal > 85%, and color removal > 97% were attained. Furthermore, membrane A85 (with 85% clay) showed the best performance, with a water flux of 659.1 L·h^−1^·m^−2^·bar^−1^. This study highlights the potential of incorporating low-cost clay material for the enhancement of the performance of mixed organic/inorganic matrix membranes, which can be applied to textile wastewater treatment.

## 1. Introduction

Membranes are increasingly used in different fields due to various advantages such as ease of processing, low power consumption, high stability, and high efficiency [1,2]. The various membrane processes using pressure as a driving force, represented by microfiltration (MF), ultrafiltration (UF), nanofiltration (NF), and reverse osmosis (RO), have given excellent results in the field of industrial effluents treatment, such as textile industry wastewater [3], electroplating industry effluent [4], and seafood conditioning effluent [5]. Usually, polymers are the main materials used to make membranes due to the advantages they offer, such as good membrane forming ability, flexibility, and low cost. MF and UF membranes primarily use polymers such as polysulfone (PSF) and polytetrafluoroethylene (PTFE) in water treatment applications [6]. However, chemical, mechanical, and thermal resistances limit the application of these polymer membranes [7].

On the contrary, the development of ceramic membranes is being studied more and more because of their interesting properties compared to organic membranes in terms of thermal and chemical stabilities [8,9] and good mechanical resistance. Furthermore, the intrinsic characteristics of the ceramic membranes correspond perfectly to the nature of textile effluents, also allowing more aggressive cleaning conditions, which increases the life of membranes [10]. However, the high sintering temperature and expensive raw materials used in the case of alumina, zirconia, titania, and silica ceramic membranes may explain their excessive prices. To overcome these limitations, researchers have turned to the use of alternative materials such as geomaterials or raw materials, which are more abundant and cheaper materials [11]. 

Natural clays have been extensively used for the preparation of low-cost ceramic membranes. This raw material has been identified as promising, since it is an indispensable nonpolluting natural material with a wide range of applications, including the polymer, water treatment, cosmetics, ceramics, paint, pharmaceutical, pulp, and paper industries [12]. In addition, clay offers high strength and low plasticity, as well as good hydrophilicity, to membranes [13]. In this regard, Abdel-Karim et al. [14] developed a cellulose acetate ultrafiltration membrane for the treatment of real hazardous textile wastewater. They proved that the incorporation of α-aminophosphonate-modified montmorillonite and silver doped with titanium oxide nanoparticles (Ag-TiO_2_NPs) into the polymer matrix allows to improve the performance, antifouling property, and durability of membranes.

Indeed, the dispersion of inorganic particles in a polymer matrix is one of the most promising and used approaches [15,16], since it offers both the advantages of ceramics and polymers. Specifically, organic–inorganic hybrid materials have the main advantages of inorganic oxides, exhibiting high mechanical, thermal, and structural stabilities, without losing the characteristics of polyvalent organic polymers, such as flexibility and functional variety [17].

It is well known from the literature that polytetrafluoroethylene (PTFE) is a highly valued polymer for its several advanced properties, such as superior thermostability, high hydrophobicity, and chemical inertness [18]. This thermoplastic polymer is considered one of the most popular polymers due to its high mechanical strength. PTFE membranes also exhibit promising filtration performance due to the formation of a cake layer membrane [19].

It has been reported that the introduction of organic functional groups on an inorganic surface sometimes contributes not only to a better dispersion of the inorganic material in polymer membranes, but also to better absorption and transportation of penetrates, which results in selectivity and favorable permeability [20,21]. Madaeni et al. [22] proved that dispersion of clay nanoparticles (NPs) in the polymer improves the performance of polymeric membranes for water treatment, such as water permeability and pollutant selectivity. Several researchers have studied the effects of the addition of clay particles on the morphology and thermal, mechanical, and hydrophilic properties, as well as on the performance, of composite membranes [23,24]. Anadão et al. [25] prepared nanocomposite membranes containing polysulfone (PSf) and sodium montmorillonite (MMT). They found that the addition of MMT is useful for producing more hydrophilic membranes in comparison to pure PSf membrane features.

Aloulou et al. [26] synthesized clay-based ultrafiltration membranes supported on a natural zeolite support. Applied to the treatment of effluents from an electroplating company contaminated with heavy metals, it was interesting to find that these membranes have good efficiency in removing a high chemical oxygen demand (COD > 96%) and a high rejection of heavy metals (Cr and Co > 80%).

In the same context, Terrazas-Bandala et al. [27] added activated carbon to cellulose triacetate to form a composite membrane very effective at removing arsenic from aqueous media. In this case, the addition of activated carbon improved the mechanical strength of the membrane, as well as allowed an increase in pore size.

Ounissi et al. [28] prepared a lithium composite membrane (LCM) for which lithium ion conductive glass–ceramic powder (LICGC) was incorporated into a polymer matrix. They showed that this membrane exhibits excellent selectivity toward lithium ions, a good mechanical resistance, and high tolerance to rigidity.

In addition, it should be noted that fouling (reversible or irreversible) is often the major obstacle to the use of the membrane processes [29]. In general, reversible fouling may be attributed to the concentration polarization and deposition of rejected substances on the membrane surface. This type of fouling can be easily removed by a simple back-flushing of the fouled membrane with water or by changing some of the operating variables such as imposing a pulsatile flow [30,31]. Irreversible fouling results from internal soiling of pores by organic and/or inorganic foulants and requires chemical cleaning to be removed [32].

Raval et al. [33] added zeolite nanopowder (in different fractions) to polysulfone polymer and made new zeolite–polysulfone composite ultrafiltration membranes. They then studied the physico-chemical and fouling performances of these new membranes and proved that (1) the optimum content is 700 mg/L of zeolite nanomaterial loading for advanced water treatment application and (2) zeolite reduces the fouling (in an albumin protein solution) of membranes and simultaneously increases their permeability. Another similar study, but focusing on biofouling [34], showed that the introduction of graphene oxide nanoparticles in polysulfone membranes makes it possible to very significantly limit the drop in performance of these nanocomposite membranes when exposed to two different bacteria (*Klebsiella* and *Pseudomonas*) for three days.

The main aims of this study were to synthesize, characterize, and use a composite polymer/inorganic membrane using low-cost and locally sourced materials such as a natural clay for the treatment of a real textile wastewater (supplied by a Tunisian textile company using indigo dye). The influence of the natural clay content on the membrane properties was studied by varying its percentage from 40 to 85 wt.% in a PTFE polymer matrix. The prepared membranes were characterized using a wide range of techniques in order to understand their chemical composition and morphological structure. Another advantage is that the membrane preparation protocol does not use any toxic or very expensive solvents or reagents.

## 2. Materials and Methods

### 2.1. Materials

The polymer used herein was polytetrafluoroethylene (PTFE; 60% vol. solution in water with a density of 2.2) purchased from Sigma-Aldrich (St. Louis, MO, USA).

Tunisian natural clay from the Tabarka region located in the northwest of Tunisia was used in this study. The sampling of this clay was taken at the top of the career of the mount Sidi El Bader, which is considered surface sampling, since it was sufficient for us to strike the ground with a hammer to recover the sample.

### 2.2. Preparation of the Composite Membranes

PTFE polymer was mixed with Tabarka clay sieved at 40 µm by simply mixing different percentages of PTFE and clay with the use of ethanol as a solvent at 70 °C to make a paste for membrane preparation.

A food rolling mill with a variable roller spacing system was used. The rolling mill had a variable roller spacing system: 18 notches or 17 intervals, allowing the thickness to be adjusted. After having kneaded the dough, we passed it through a rolling mill, spacing the dough as much as possible in the rollers. The dough was spread by the rollers, and then we reduced the play step-by-step in order to avoid the formation of balls at the smallest thickness and while changing with each pass the direction of application of the roller throughout the preparation, which provided a membrane surface without cracks.

In this case, the organic and inorganic compounds were integrated together without phase separation, being uniformly distributed in the network. These organic–inorganic materials can only be achieved by establishing a stable connection between organic and inorganic groups [17,35].

Three membranes were made with different percentages of clays and PTFE. The membranes A85, A50, and A40 contained percentages of clay of 85%, 50%, and 40%, respectively.

### 2.3. Powder Characterization

The clay powder was taken from the area of Tabarka (northwest Tunisia) and was characterized using different techniques. The mineralogical composition was determined by XRD analysis, while the chemical compositions were obtained after X-ray fluorescence. Thermogravimetric analysis (TGA) and infrared analysis were carried out. The clay fraction with a diameter of less than 2 μm was measured by laser particle sizing using a Mastersizer Malvern particle sizer. The sample was dispersed in deionized water, and the percentage of clay particles < 2 μm was 44%. All details of this powder characterization are given by Felhi et al. [36].

### 2.4. Membrane Characterization

Analogical palmer for the Käfer brand was used with a measuring range from 0 to 1000 µm for the determination of the different membrane thicknesses.

The hydrophilic/hydrophobic character of the membranes was determined by measuring the contact angle of a distilled water drop on the membrane surface (optical contact angle model DSA100, KRUSS Co., Kruss, Germany). Ten different points were considered for the sample. The mean value of the contact angles was then reported.

The morphology of each membrane was investigated using scanning electron microscopy (SEM) (MERLIN scanning electron microscope by ZEISS associated with a GEMINI II column, Göttingen, Germany). The surface elemental composition of the samples was also analyzed using an energy dispersion X-ray (EDX) fitted to the SEM equipment. Infrared absorption spectra were recorded using a Perking-Elmer Spectrum BX FI-IR device.

To determine the mechanical property of the membranes, tensile tests were performed to determine the Young’s modulus (E) and tensile stress (TS). A two-column tensile machine (INSTRON 5965, Norwood, Massachusetts, USA) associated with a tensile load cell with a capacity of 100 N at a tensile speed of 1 mm·min^−1^ was used.

Measurement of the water uptake (*WU*) was determined from the difference in weight between the dried and the swollen membranes. The membrane samples were dried under vacuum at a fixed temperature of 60 °C until a constant dry weight was obtained. They were then soaked in water for 24 h. Before weighing, any excess water on the membrane surface was removed by adsorbent paper. The percentage water content of the membrane was calculated using Equation (1):

(1) $$WU=100\times \frac{W_{1}-W_{2}}{W_{2}}$$

where *W*_1_ and *W*_2_ are the weight of the wet and dry membranes, respectively.

The membrane pore diameter size was determined using the differential scanning calorimetry (DSC) method according to the study of Kononenko et al. [37]. DSC analyses were carried out on a TA Instrument Q25 DSC, which was designed to directly quantify the enthalpy changes of a heat-treated sample. This instrument was operated in a temperature range of −80 to 450 °C in an atmosphere of nitrogen, with a heating/cooling rate of 0.01–100 °C·min^−1^.

Thermogravimetric analyses (TGAs) were conducted on a SeteramSetsys Evolution 16 apparatus. Samples were heated from 20 to 40 °C at 10 °C·min^−1^ and kept at 40 °C for 10 min for stabilization, then heated from 40 to 1100 °C at 10 °C·min^−1^ under an air atmosphere. Finally, they were cooled from 1100 to 20 °C at 30 °C·min^−1^ and kept at 20 °C for 30 min.

### 2.5. UF Experiments

**Figure 1:**
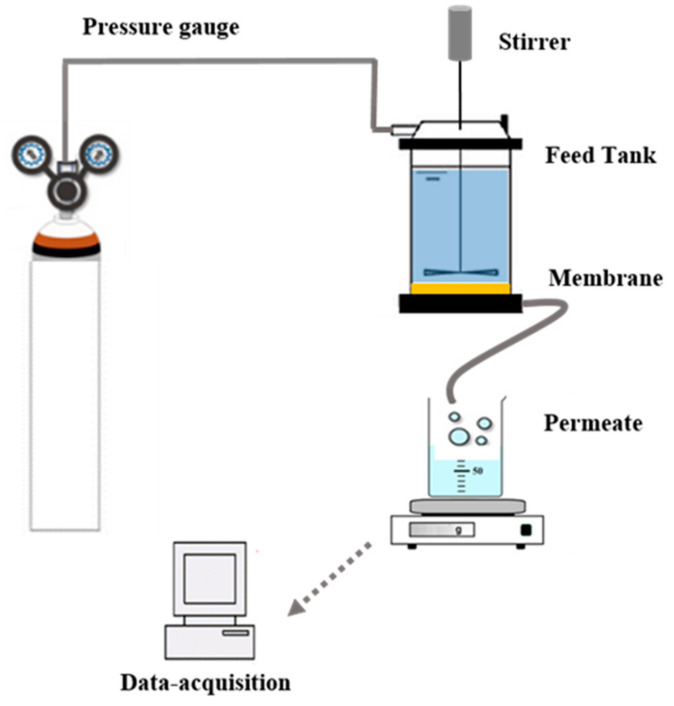
Illustration of the experimental setup for treating the wastewater.

The application of the prepared membranes to wastewater treatment was carried out using a homemade filtration setup (Figure 1). After having placed the flat sheet circular membrane (90 mm in diameter) in the filtration cell, the cell was filled with the solution to be filtered. The agitation was set at 350 rpm. Nitrogen gas was bubbled into the filtration cell to adjust the pressure in the cell to 2.5 bar, 5.0 bar, and 7.5 bar. The permeate flux (in L·h^−1^·m^−2^) was determined from the time required to collect a given volume of permeate per unit of membrane surface area. The removal efficiency of the membrane in terms of COD, turbidity, and color was evaluated using Equation (2):

(2) $$R(\%)=100\times (1-\frac{C_{p}}{C_{f}})$$

where *C_f_* and *C_p_* are the concentrations in the feed and permeate, respectively, and *R* is the removal efficiency expressed as a percentage.

### 2.6. Fouling Study

After a filtration run, the membrane was rinsed with distilled water and the tank was filled with 90 mL of distilled water afterward. For the membrane regeneration, only distilled water was used at a pressure of 2.0 bar without adding any cleaning agents. The duration of each regeneration cycle was 20 min, followed by determination of the membrane resistance using Equation (4).

In this study, the membrane fouling was evaluated using the flux recovery ration (*FRR*) (given by Equation (3)) and the resistance-in-series model [38,39,40] (see Equation (4)).

(3) $$FRR=100\times \frac{J_{w1}}{J_{w0}}$$

where *J_w_*_0_ is the water permeate flux of the unused membrane and *J_w_*_1_ is the water permeate flux of the membrane obtained after a simple rinsing with distilled water.

(4) $$R_{T}=R_{m}+R_{rev}+R_{irr}$$

where *R_m_* is the inherent hydraulic resistance (m^−1^) due to the membrane, obtained from the water permeability test; *R_irr_* is the resistance due to irreversible pore soiling, determined after rinsing and measurement of the flow rate with distilled water (m^−1^); and *R_rev_* is the resistance due to reversible fouling (m^−1^).

## 3. Results

### 3.1. Clay Characterization

**Figure 2:**
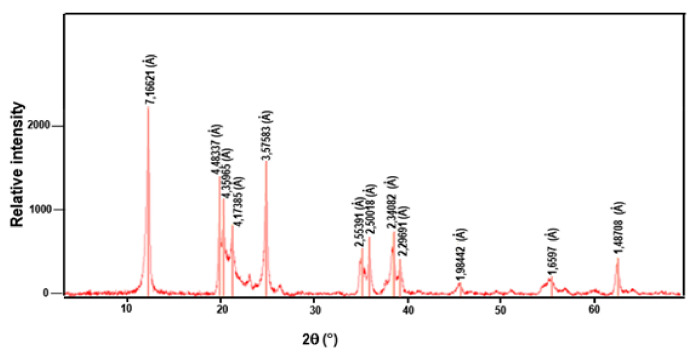
X-ray diffraction diagram of the Tabarka clay.

The chemical composition of the Tabarka clay in weight percentages of the various oxides was obtained by an X-ray diffraction diagram (Figure 2) and is given in Table 1. This table reveals that the clay powder was mainly composed of a large quantity of silica SiO_2_ and alumina Al_2_O_3_. The loss of ignition (LOI) was 9.8% due to H_2_O loss from the clay minerals on the one side, and decarbonation on the other. In addition, the presence of other alkaline earth oxides, such as CaO, SO_3_, and MgO, at lower amounts can be considered acceptable for the preparation of ceramic membranes. The presence of a high SiO_2_ content in the analyzed sample can also be explained by the existence of quartz, as shown by the diffraction peak. Moreover, the mineralogy analysis showed the presence of kaolinite (61%) and illite (39%) minerals, while the non-clayey minerals was essentially represented by quartz.

**Figure 3:**
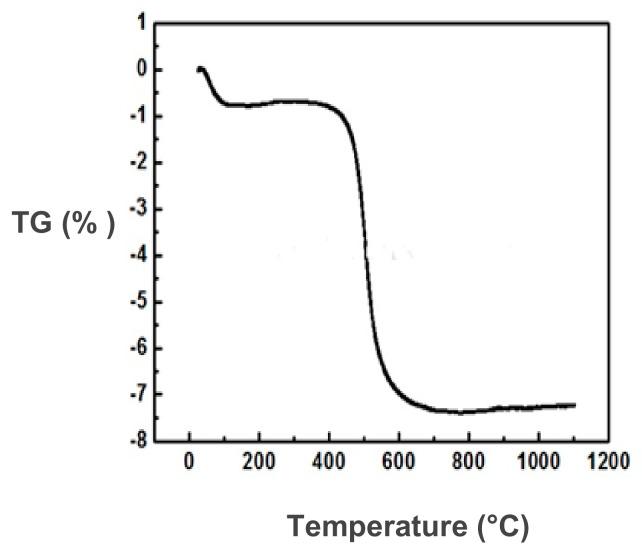
TGA analysis for Tabarka clay.

It can be observed that the form of the TGA curve is characteristic of kaolinite minerals (Figure 3). Four main peaks can be distinguished; the first, an endothermic peak, is located between 100 and 150 °C and corresponds to dehydration, and, from the TGA analysis, it is approximately 0.76%, corresponding to the release of adsorbed water. The second peak is located at 530 °C and corresponds to the dehydroxylation of the clay mineral. A mass loss of approximately 4% due to the destruction of kaolinite was observed. The third peak is an endothermic peak due to the decarbonation, obtained at almost 800 °C. The last one is an exothermic peak observed at approximately 960 °C and corresponds to the phase transformation of illite–metakaolinite–mullite. This transformation did not engage any mass loss, and the total mass loss retained the value of 7.5%.

### 3.2. Membrane Characterization

#### 3.2.1. FTIR

**Figure 4:**
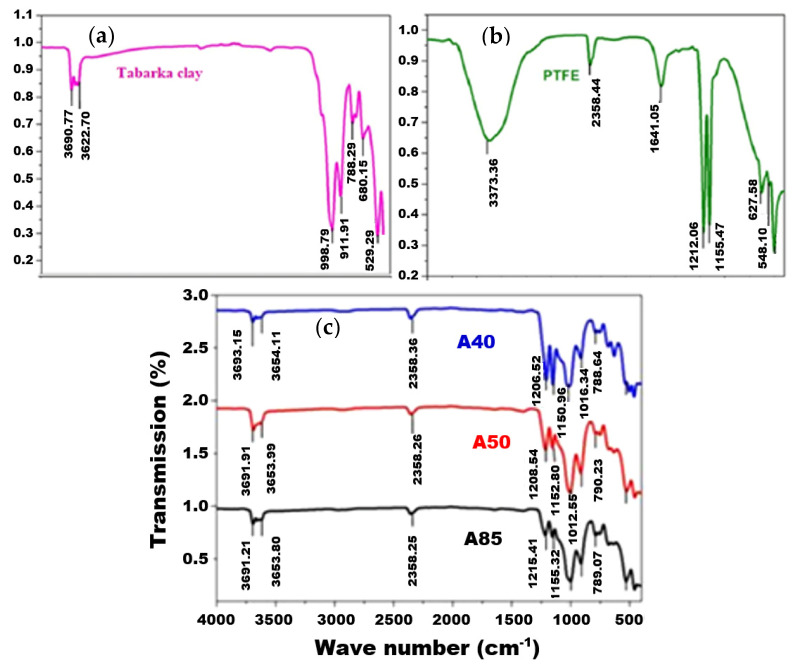
FTIR spectrum of pure clay (**a**), pure PTFE (**b**), and composite A40, A50, and A85 membranes (**c**).

Figure 4 shows a comparison between the FTIR spectrum of the pure clay (a), the pure PTFE (b), and the A50 composite membrane (c). The pure clay shows the following characteristic vibration bands: Si-O (998.79 cm^−1^), Si-O-Al (529.29 and 788.29 cm^−1^), Al-OH (911.91 cm^−1^), and OH (3690.77 and 3622.70 cm^−1^) [41,42]. On the contrary, the spectrum of pure PTFE shows bands at 1212.06 cm^−1^ and 1155.47 cm^−1^ attributed to the symmetrical stretch of CF_2_, a band at 627.58 cm^−1^ corresponding to the deformation of CF, and a band at 548.10 cm^−1^ attributed to the symmetrical strain of CF_3_ [43].

The comparison of the FTIR spectra of the pure PTFE, the clay, and the composite membranes (A85, A50, and A40) suggests that the characteristic bands of PTFE and clay are present in the different prepared membranes. Indeed, for membrane A50, for example, both bands located at 12,028.54 and 1152.80 cm^−1^ mark the presence of CF_2_. The band that appeared at 1012.55 cm^−1^ is assigned to the bond vibration of Si-O, and the two bands recorded at 3691.91 and 3653.99 cm^−1^ are characteristic of the OH group. This last result confirms that the OH group remained clear and therefore did not participate in the establishment of polymer–clay bonds and that the composite membrane was obtained thanks to the establishment of hydrophobic bonds linked to adsorption and to the change in the ionic environment. These results are widely observed in the literature [26,44,45] and are attributed to class I hybrids in which organic and inorganic materials are normally linked by means of Vander Waals bonds, hydrogen bonds, or a simple physicochemical adsorption [46].

#### 3.2.2. Mechanical Propriety

Adequate mechanical strength is also an important factor for membranes. The tensile strength of the membranes was examined at room temperature in a dry state and the results are gathered in Table 2.

The results show that both the Young’s modulus and tensile strength increased proportionally with the increase in clay content, from 10 and 0.41 MPa for the A40 membrane to 24 and 2.32 MPa for the A85 membrane for the Young’s modulus and maximum tensile strength, respectively. In the same context, Uthirakumar et al. [47] reported a 50% improvement in the Young’s modulus of a polystyrene film reinforced with 5 wt.% of clay. Hwang et al. [48] observed an improvement in mechanical strength when adding clay to obtain PVDF/clay composite membranes. These results are in agreement with the study developed by Lewandowska et al. [49], indicating the effectiveness of adding montmorillonite (MMT) to chitosan to improve mechanical strength.

Clay is a mineral filler that is very hydrophilic and much more resistant than PTFE. Thus, we introduced it in large quantities (85 wt.%) and after solidification by evaporation of the solvent (water), this clay created a more rigid (high value of E) and quite resistant (high value of maximum tensile strength) membrane. Moreover, we observed a clear correlation between the clay contents and the values of E and TS.

#### 3.2.3. Membrane Morphology Observation

**Figure 5:**
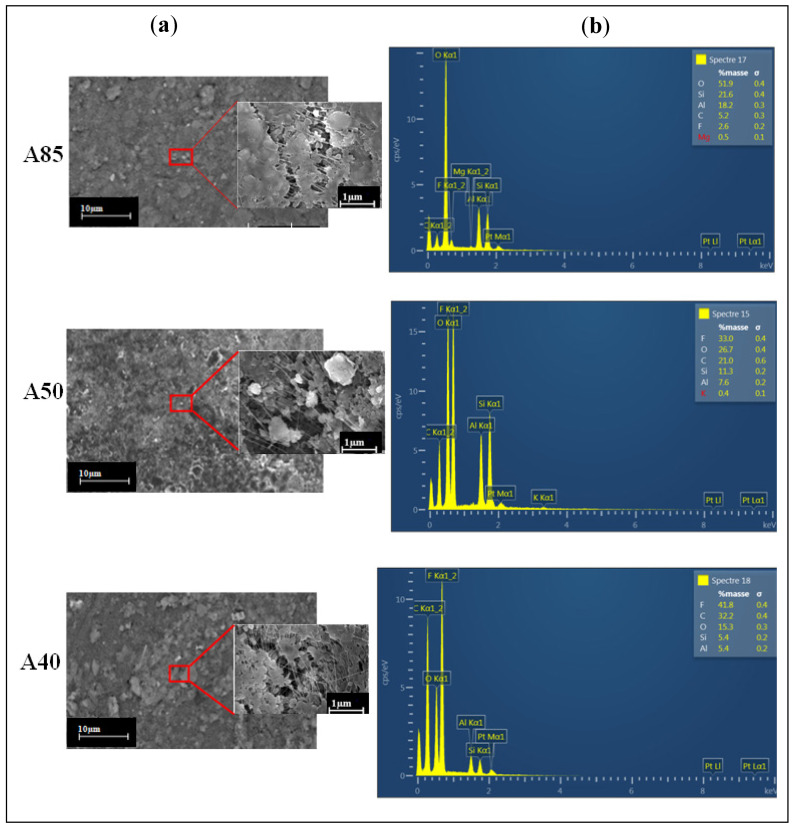
SEM surface images (**a**) and EDX spectra (**b**) of the three studied membranes.

In order to analyze the effects of adding clay to the PTFE polymer matrix on the membrane morphology, SEM surface images and EDX analysis were performed (Figure 5).

Figure 5a shows that the aggregated particles were evenly distributed over the outer surface of the composite membranes, responsible for the surface roughness [50]. This is associated with the fact that this behavior contributes to the formation of pores on the surface of composite membranes [51]. Moreover, we can conclude that the formation of a porous structure came from the penetration and intercalation of PTFE polymer in the layers of the clay. Rekik et al. [52] reported that a well-defined porous structure can provide better conditions to improve the permeability.

EDX spectra analysis for the A85, A50, and A40 membranes (Figure 5b) clearly shows the presence of F and C chemical elements (indicating the presence of PTFE), as well as the relative signals of Si, Al, and O (characteristics of clay). The distribution of these elements confirms the formation of clay/PTFE composite membranes.

As can be expected, the A85 membrane had the highest Si, Al, and O contents compared to the others (A50 and A40), which were 21.1%, 18.2%, and 51.9%, respectively, and the lowest F (2.6%) and C (5.2%) contents.

**Figure 6:**
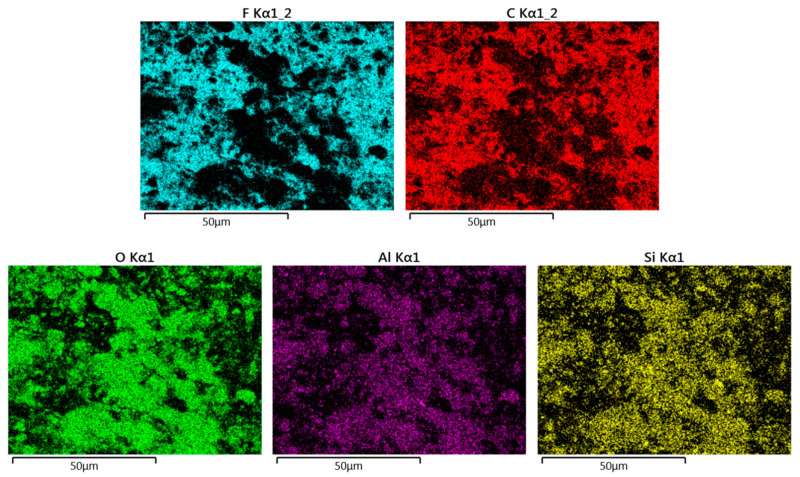
EDX mapping images representing the distributions of F and C (elements of PTFE polymer) and O, Al, and Si (elements of the clay matter) in the A50 membrane.

The A50 membrane can be considered a representative example of all membranes. Its X-mapping given by Figure 6 shows that its fluorine and carbon atoms (constituting the PTFE polymer) were in the same place, and that aluminum, silicon, and oxygen atoms (elements of clay matter) were in complementary places. All of these elements prove that the two phases (PTFE and clay) were well juxtaposed, without separation or segregation, and occupied the whole volume of the membrane.

#### 3.2.4. Determination of Pore Diameters

**Figure 7:**
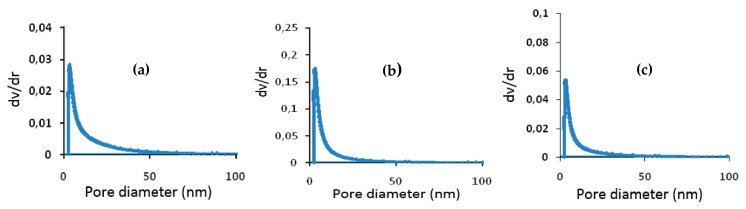
Distribution of pore diameters for the composite membranes (**a**) A85, (**b**) A50; (**c**) A40.

Figure 7a–c illustrate the pore diameter for the three composite membranes A85, A50, and A40: 8 nm for A85 membrane, 7 nm for A50 membrane, and 6 nm for A40 membrane. Meanwhile, in the work of Bousbih et al. [52], the preparation of a 100% clay monolayer membrane sintered at 1000 °C showed a pore size of 35 nm. This result indicates a decrease from 35 nm for the totally clay membrane to (8–6 nm for the composites of the A85, A50, and A40 membranes. This behavior could be associated with the dominance of the polymer matrix responsible for the reduction of the pore size in comparison to the membrane made entirely of clay. The pore type for the three membranes was mesoporous.

**Figure 8:**
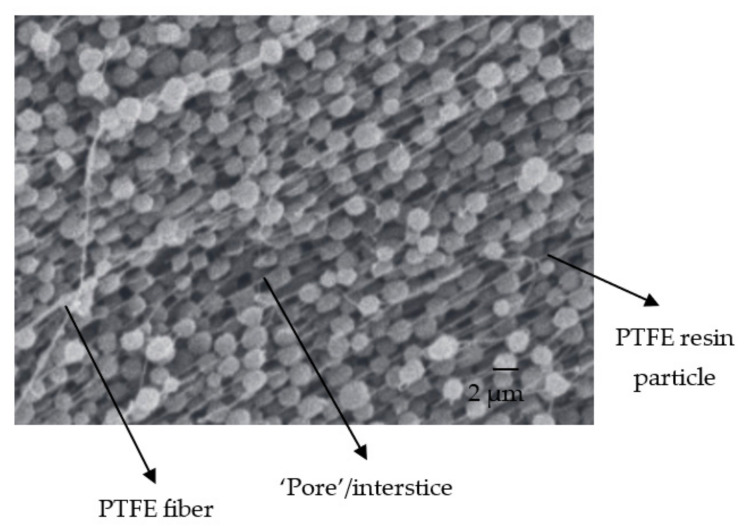
SEM image of fibrilled PTFE resin particles. Adapted from [53].

The procedure we chose to prepare our membranes (see Section 2.2) consists of promoting fibrillation of the PTFE resin particles, which are, on average, 1–2 µm in diameter, to ensure their cohesion. These are the fibers that appear in some of the SEM images in Figure 5a, and are even clearer in Figure 8, adapted from the work of Ardakani et al. [53]. The appearance of these fibers and the mechanical compression during the shaping of the membranes considerably reduced the free interstices for the passage of the different molecules, such as water molecules. Thus, we obtained very thin "pores" with a diameter of approximately 10 nm. The introduction of clay particles (typically 1–50 µm in diameter) slightly increased the interstices either by the creation of microdefects (see Figure 5a), by the breaking of some fibers, or by a decrease in their number. Moreover, we observed that in the absence of PTFE (100% clay), the “pore” diameter was around 35 nm.

#### 3.2.5. Determination of Thickness, Water Uptake, and Contact Angle of Membranes

The thickness of the prepared membranes varied between 362 and 432 µm. The addition of clay did not seriously affect the thickness, but did affect the contact angle, which decreased from 87° for membrane A40 to 70° for membrane A85 (Table 3).

The thickness of the membrane depends mainly on the ability of the laminator to spread the PTFE and clay. It also depends on the number of times the patch is passed through the rolling mill. This number is not necessarily the same from one membrane to another, and the random geometry of the membrane following rolling means that the thickness is not well controlled. There is no relationship between this thickness and the initial composition of the mixture.

The measurements of the contact angle and the swelling rate (water uptake) show that for membrane A85 (where the percentage of clay was the highest), there was a higher swelling rate and a smaller contact angle (70°). The contact angle decreased with the increasing clay content, indicating that addition of clay can be a useful means of improving the hydrophilicity of the polymeric membrane, in agreement with Anadão et al. [25]. Thus, increasing the level of PTFE allows the swelling rate to be reduced. This was corroborated by the fact that membrane A40, containing the highest percentage of PTFE, displayed the highest contact angle. For instance, pure PTFE had a large contact angle of 115 ± 8.5° [54,55].

The introduction of clay to the polymer allowed the acquisition of a new structure, where the organic and inorganic components were linked together by strong chemical bond, resulting into the modification of the surface property of the polymer. In particular, the introduction of organic groups into an inorganic network can provide various tunable properties such as flexibility, solubility, and hydrophobicity [17,56,57]. In addition, the literature shows that the adsorption–desorption capacities of a polymer could be enhanced due to the combination of hydrophobic and hydrophilic sub-domains [17].

### 3.3. Application to the Treatment of Real Textile Effluent by UF

#### 3.3.1. Characterization of the Textile Effluent Used

The performance of the produced membranes was evaluated using textile wastewater from a Tunisian textile factory with a focus on the removal of indigo as a dye. Table 4 presents the physicochemical characteristics of the raw effluent.

#### 3.3.2. Determination of the Water Permeability 

**Figure 9:**
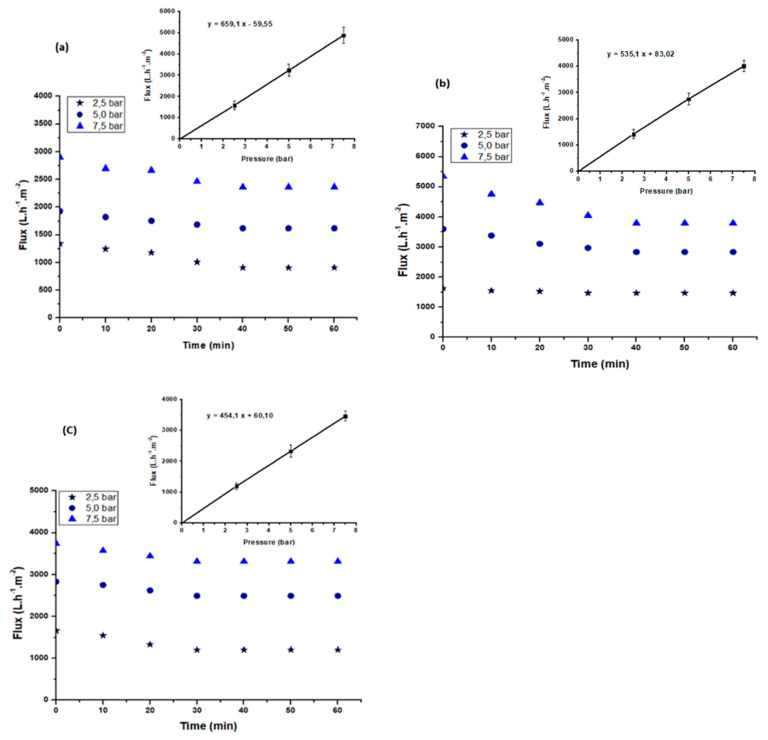
Variation of the permeate flux of the textile effluent with time at different pressures for the A85 (**a**), A50 (**b**), and A40 (**c**) membranes.

Steady-state membrane flux at different pressures (from 2.5 to 7.5 bar) was obtained after 30 min of filtration for each composite membrane, i.e., A85, A50, and A40 (Figure 9a–c). Three measurements were taken and the error was calculated. The calculated error was between 5% and 15%. The water permeability of each membrane corresponds to the slope of the straight line obtained following the determination of the permeate flux as a function of the pressure according to Darcy’s law [58].

Consequently, the permeability of the membranes was calculated as 659 L·h^−1^·m^−^2·bar^−1^, 535 L·h^−1^·m^−^2·bar^−1^, and 454 L·h^−1^·m^−^2·bar^−1^ for A85, A50, and A40, respectively. The results reveal that the permeability of the A85 membrane, which had the largest amount of the clay, was the highest of the three membranes. This result is consistent with that obtained by Mierzwa et al. [59]. The authors reported that the permeability of the polyethersulfone ultrafiltration membrane increased with the increase in the amount of clay. Furthermore, the authors reported an increase in the permeability of the membrane from 312 L·h^−1^·m^−2^·bar^−1^ to 389 L·h^−1^·m^−2^·bar^−1^ when the quantity of the clay was increased from 1% to 2%.

#### 3.3.3. Effect of Transmembrane Pressure

The filtration performance is generally evaluated by the determination of the permeate flux and the selectivity of the membrane in terms of the removal ion rate of the different pollutants. This is particularly required to evaluate the performance of a membrane using real wastewater effluent. The use of the different composite membranes to treat real textile effluent was carried out at ambient temperature and at various pressures of 2.5, 5.0, and 7.5 bar. Figure 9a–c show the evolution of the permeate flux with time at different transmembrane pressures (TMPs) for the three membranes—A85, A50, and A40. The permeate flux decreased with time to stabilized after 30 min of filtration at 263, 316, and 361 L·h^−1^·m^−2^, respectively, for the A40, A50, and A85 membranes. The decrease in flux with time was due to the deposition of suspended particulate matter and macromolecules on the membrane surface, leading the formation of a porous cake that was more or less compact depending on the pressure value.

On the contrary, it can be observed that the permeate flux increased linearly with the pressure. This behavior indicates that the mass transfer in this case was controlled by convection.

The different removal rates of pollutants (turbidity, conductivity, COD, and color) at the different pressures was determined and are reported in Table 5. For all membranes, an almost total removal of the turbidity was observed. COD removal was between 81% and 82% and that of color removal was between 95% and 96%. These results indicate that the retention was independent of the composite membrane composition. However, the performance decreased with the pressure due to the passage of macromolecules through the membrane pores.

Therefore, it can be concluded that at a transmembrane pressure of 2.5 bar, our membranes displayed a performance that indicates the almost total removal of turbidity, >97% color removal, and >85% COD removal.

According to these results, despite the different compositions of the clay/PTFE membranes, the performance was almost the same for the three membranes. Additionally, the pH and the conductivity remained almost unchanged after the filtration.

Based on these results, a comparison can be made between the performance of these composite membranes and that of the membrane made totally from clay that was previously developed and tested in our laboratory [52]. A similar behavior could be deduced for the membrane in [52] at an optimal pressure of 5.0 bar [52] and the membrane in this article when tested at 2.5 bar. It could be assumed that achieving a similar performance at an approximately 50% reduction in operating pressure with the membrane in this present study could translate into a significant reduction in energy consumption during filtration. In another report, Derouich et al. [60] developed a UF membrane based on polypyrrole/pozzolan, which eliminated more than 98% of Congo red dye from a wastewater.

#### 3.3.4. Fouling Study

**Figure 10:**
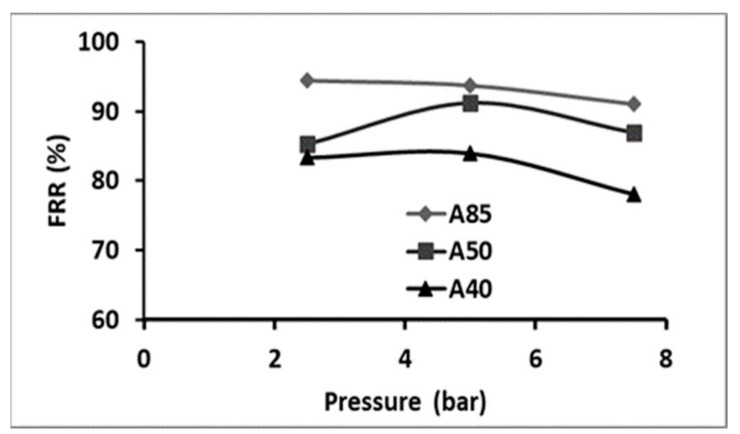
Evaluation of the FRR with pressure for the different composite membranes.

Fouling is one of the main problems in membrane separation processes, which is caused by dissolved organic or inorganic compounds, colloids, suspended solids, bacteria, etc. Nevertheless, for nanofiltration and reverse osmosis, it might be even more complex due to the interaction between the solutes and the membrane surface, leading to membrane fouling, which can take place at the nanoscale [61,62]. The FRR is a criterion indicating the fouling behavior of a membrane and it is preferable to be closer to 100%. The membrane fouling was estimated by determining the flux recovery rate (FRR) at various pressures (Figure 10). A decrease in FRR was observed with the increase in pressure, starting from a pressure of 5 bar. The highest FRR was obtained at 2.5 bar for membrane A85. A slight decrease in the FRR was observed when the content of polymer increased. At the optimal TMP of 2.5 bar, the value of FRR was of 94.4%, 85.3%, and 83.3% for the A85, A50, and A40 membranes, respectively (Figure 10).

To explain the fouling behavior of the membrane with respect to a decline in the permeate flux with time, the resistance in the series model was used. The different calculated resistance values for the different membranes are summarized in Table 6.

Table 6 shows that reversible fouling was the dominant mechanism during filtration for the three prepared membranes (*R_irrev_* ˂ *R_rev_*). Therefore, just a simple rinsing with water was needed for membrane regeneration.

Listed in Table 7 are the performances of some recent composite membranes and two other polymer membranes, all used for the dye effluent treatments. Other membranes optimized for dye/mineral salt separation are not given in this table. We remark that our A85 membrane had better performance than most of these membranes, distinguished by high permeability and a good dye removal rate, allowing a fast treatment of the effluents generally in only one step.

#### 3.3.5. Membrane Regeneration

The regeneration of the composite membranes was carried out by a simple method, whereby the membrane was rinsed with distilled water after each use. This procedure was repeated for different cycles. The efficacy of the regeneration method was checked via the comparison of water permeability of the used membrane with that of the unused membrane. Ideally, for 100% regeneration, the original water permeability (membrane flux) of the unused membrane should be attained.

**Figure 11:**
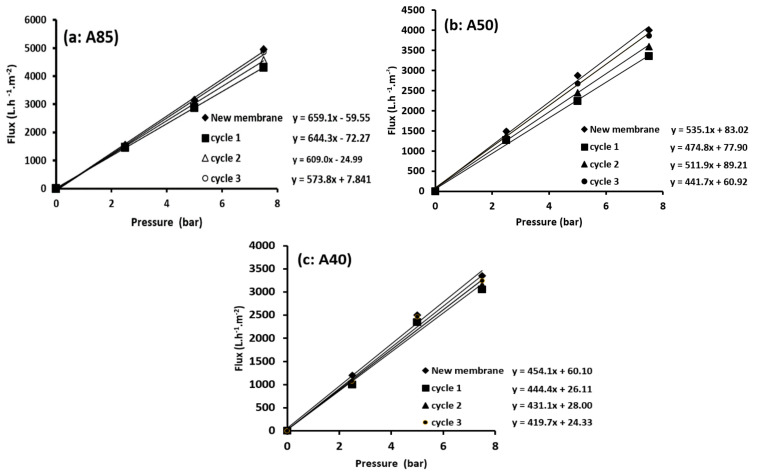
Regeneration tests of the A85 (**a**), A50 (**b**), and A40 (**c**) membranes.

From Figure 11a–c, a slight decrease in performance after the second cleaning cycle was detected. Almost complete regeneration of the A85, A50, and A40 membranes was observed after three cycles. This behavior was already observed by Khemakhem et al. [69] when a ceramic UF membrane made from mud (sub-product from phosphate industry transformation) was tested.

## 4. Conclusions

In this study, three PTFE/clay composite membranes with 85 wt.% (A85), 50 wt.% (A50), and 40 wt.% (A40) of clay were prepared. The effect of the clay content on the physical, chemical, and mechanical properties of the composite membranes was studied. It was observed that the addition of clay improved the porous structure, the mechanical strength, and the hydrophilicity of the membranes. The enhanced hydrophilicity of the membranes resulted in an improvement in the membrane permeability. 

Performance evaluation of these membrane in removing azo dye from textile industry wastewater revealed that the A85 membrane, which contained more clay, displayed the best performance, with a water permeability of 659 L·h^−1^·m^−2^·bar^−1^. In addition, the best performance achieved at a transmembrane pressure of 2.5 bar showed that the permeate contained less turbidity (<1 NTU), less color (approximately 97% removal), and less COD (approximately 85% removal). Finally, the tests of membrane regeneration confirmed that the potential application of a hybrid PTFE/clay membrane materials as a UF membrane for treating textile wastewater.

## Figures and Tables

**Table 1:** Chemical composition of Tunisian clay from the Tabarka region (wt.%).

Constituents	SiO_2_	Al_2_O_3_	CaO	Fe_2_O_3_	MgO	Na_2_O	K_2_O	SO_3_	LOI ^1^
Wt.%	55.25	24.17	0.16	1.15	5.39	0.19	1.78	0.40	9.77

^1^ LOI, loss on ignition.

**Table 2:** Measurements of mechanical resistance.

Membrane	E: Young’s Modulus (MPa)	TS: Maximum Tensile Strength (MPa)
**A85**	24	2.32
**A50**	18	0.82
**A40**	10	0.41

**Table 3:** Same physical properties of the composite membranes (clay/PTFE).

Membrane	Membrane Thickness (µm)	Water Uptake (*WU*)	Contact Angle (°)
**A85**	383 ± 2	36	70 ± 2
**A50**	432 ± 2	30	81 ± 3
**A40**	362 ± 2	25	87 ± 3

**Table 4:** Physicochemical characteristics of the textile effluent used.

Sample	pH	Conductivity (mS·cm^−1^)	Turbidity (NTU)	COD	Abs. at λ_max_ = 590 nm
**Raw effluent**	12.5	6.2	576	2075	1.836

**Table 5:** Characteristics of the effluent before and after filtration using the different membranes.

Sample		pH	Conductivity (mS/cm)	Turbidity (NTU)	COD	Abs at λmax = 590 nm
**Raw effluent**		12.5	6.2	576	2075	1.836
**TMP = 2.5 bar**	A40	12.3	4.3	0.80	301	0.051
	A50	12.4	4.0	0.70	264	0.043
	A85	12.58	3.9	0.58	287	0.041
**TMP = 5.0 bar**	A40	12.39	4.7	0.82	347	0.061
	A50	12.45	4.4	0.76	359	0.066
	A85	12.54	4.3	0.51	384	0.045
**TMP = 7.5 bar**	A40	12.41	5.0	0.91	391	0.085
	A50	12.4	4.5	0.80	380	0.073
	A85	12.49	4.4	0.60	405	0.067

**Table 6:** Determination of the different values of the resistances according to the series resistance model.

Sample	*R_T_* 10^12^ (m^−1^)	*R_m_* 10^12^ (m^−1^)	*R_rev_* 10^12^ (m^−1^)	*R_iri_* 10^12^ (m^−1^)
**Membrane A85**	0.12	0.54	0.62	0.010
**Membrane A50**	0.99	0.67	0.24	0.076
**Membrane A40**	1.50	0.72	0.77	0.013

**Table 7:** Comparison of the performance between the PTFE/clay UF membrane and some reported membranes.

Membrane	Permeability (L·h^−1^·m^−2^·bar^−1^)	Color Removal (%)	COD (%)	Operation Mode	Reference
**PES**	40	98	61	Cross-flow	[63]
**PI**	345	98–65	-	Dead-end	[64]
**PVA-B-Ph/TiO_2_**	33	80–98 ± 2	-	Cross-flow	[65]
**Multichannel tubular TiO_2_-ZrO_2_**	-	62–79	62–79	Cross-flow	[66]
**Nano-TiO_2_-clay-alumina membrane**	117–850	99	-	Cross-flow	[67]
**PSF/PEI-pozzolan**	24.2	96.1–75.8	-	Cross-flow	[68]
**A85 (PTFE/clay)**	659	97	85	Dead-end	This work

## Data Availability

Not applicable.
